# On the Canada Leprosy

**Published:** 1845-02

**Authors:** James B. Johnston

**Affiliations:** Sherbrooke, Canada East


*On the Canada Leprosy.*—To the Editor of the "Medical Times."—Sir,—In the Medical Times, for the 25th of May last, you express a wish that some of your Canadian subscribers will will communicate with you, on the subject of the new malady, which has appeared in the district of Tracadia, province of New Brunswick, on this continent. In case no one else has written to you about the matter, I will state a few particulars. The government appointed a commission of medical men, in March last to visit Tracadia and the adjoining districts, and to investigate the nature of the malignant disease, reported to prevail therein. Dr. Skene, assistant surgeon of the 52d Regt., stationed at Fredericton, N. B., one of the commissioners, has made a report on the disease, addressed to Sir J. Macgrigor, Director General of the Army Medical department; from which it appears, that this complaint is unquestionably tubercular leprosy, or the Elephantiasis of the Greeks; that the symptoms of all the cases which existed at that time, nineteen in number, corresponded with those of the *lepra tuberculosa* of Bateman, Simpson, Copland and others. The symptoms observed were: dusky red or livid tubercules, of various sizes on the face, ears, and extremities; thickened or rugous state of the skin, diminution of its sensibility; falling off of the hair of the eyebrows, eyelids, and beard; voice husky, nasal or entirely lost; ozæna; ulcerations of the surface; breath intolerably fœtid; little or no pain: the nose, lips, and ears, generally enlarged and pendulous; the skin shining as if smeared with oil; the palate and fauces covered with tubercles; ulcerations and blotches on various parts of the body; appetite usually unimpaired; and the disease hitherto invariably fatal, at the end of a few years. Dr. Skene considers the disease identical with the tubercular leprosy, which prevailed in Europe, in the middle ages and more recently in Ireland, the Farroe Islands, Shetland, Madeira, the Crimea, Africa, Ceylon, and the East and West Indies. The new locality of this disease forms a part of the province of New Brunswick, and is chiefly confined to the east side of the land, lying between the bay of Chaleur and the estuary of the Miramichi river, and more particularly to the settlements on the Neguac and Tracadia rivers;—From the statements of the oldest inhabitants, the first case occurred about fhe year 1817, in the person of a woman named Ursale Landre; she died of the disease in 1829; her husband took the disease three or four years before her death, and sunk under it in 1831. From these cases, the disease would appear to have gradually extended itself, and although, ten or eleven years ago, only two cases existed, the commissioners found, independently of twelve deaths from the leprosy, nineteen confirmed cases and some highly suspicious ones. The disease appears to be transmitted by hereditary taint and by contagion, which latter is by no means active, as all those brought into direct contact with the disease, and all those immediately connected with the sources of the malady, do not necessarily become affected by it. So that it is not at all likely that this disease, which is at present local or endemic, will ever become epidemic. As to the causes of the present leprosy, in New Brunswick, it is attributed by some to filth, indigence, exposure to extreme temperatures, scanty and unwholesome diet, parricularly of fish, salted while in a state of decomposition. The commissioners recommend to the Government of New Brunswick, the erection of a lazaretto, strict seclusion of the lepers in this establishment, and legislative sanction for the removal of persons afflicted into it.

I have the honor, to be, sir,
Your most obedient servant,
JAMES B. JOHNSTON, M. D.

*Sherbrooke, Canada East, August 17th,* 1844

